# Flexible Electrospun PVDF/PAN/Graphene Nanofiber Piezoelectric Sensors for Passive Human Motion Monitoring

**DOI:** 10.3390/s26020391

**Published:** 2026-01-07

**Authors:** Hasan Cirik, Yasemin Gündoğdu Kabakci, M. A. Basyooni-M. Kabatas, Hamdi Şükür Kiliç

**Affiliations:** 1 Department of Physics, Faculty of Science, Selçuk University, Konya 42075, Turkey; 2 Laser Induced Proton Therapy Application and Research Center, Selçuk University, Konya 42075, Turkey; 3 High Technology Research and Application Center, Selçuk University, Konya 42075, Turkey; 4 Department of Precision and Microsystems Engineering, Delft University of Technology, Mekelweg 2, 2628 CD Delft, The Netherlands; 5 Institute of Nanotechnology, Karlsruhe Institute of Technology, Kaiserstraße 12, 76131 Karlsruhe, Germany; 6 Department of Nanotechnology and Advanced Materials, Graduate School of Applied and Natural Sciences, Selçuk University, Konya 42030, Turkey; 7 Department of Metallurgical and Materials Engineering, Faculty of Engineering, Dokuz Eylül University, Buca, Izmir 35160, Turkey

**Keywords:** electrospinning, PVDF, graphene, flexible piezoelectric sensor, human motion monitoring

## Abstract

Flexible piezoelectric sensors based on electrospun poly(vinylidene fluoride) (PVDF)/polyacrylonitrile (PAN)/graphene nanofibers were fabricated and evaluated for passive human body motion detection. Optimized electrospinning yielded smooth, continuous fibers with diameters of 200–250 nm and uniform films with thicknesses of 20–25 µm. Fourier transform infrared (FTIR) spectroscopy confirmed a high fraction of the piezoelectrically active β-phase in PVDF, which was further enhanced by post-deposition thermal treatment. Graphene and lithium phosphate were incorporated to improve electrical conductivity, β-phase nucleation, and piezoelectric response, while PAN provided mechanical reinforcement and flexibility. Custom test platforms were developed to simulate low-amplitude mechanical stimuli, including finger bending and pulsatile pressure. Under applied pressures of 40, 80, and 120 mmHg, the sensors generated stable millivolt-level outputs with average peak voltages of 25–30 mV, 53–60 mV, and 80–90 mV, respectively, with good repeatability and an adequate signal-to-noise ratio. These results demonstrate that PVDF/PAN/graphene nanofiber films are promising candidates for flexible, wearable piezoelectric sensors capable of detecting subtle physiological signals, and highlight the critical roles of electrospinning conditions, functional additives, and post-processing treatments in tuning their electromechanical performance.

## 1. Introduction

Recent progress in wearable electronics, personalized health monitoring systems, and medical diagnostic devices has created a strong demand for flexible, highly sensitive, and biocompatible sensors [1,2]. Among the different transduction mechanisms, piezoelectric sensors, which convert mechanical stimuli into electrical signals, show great promise for real-time monitoring of physiological parameters such as arterial pulse, respiration, muscle activity, and joint motion [3,4,5].

Poly(vinylidene fluoride) (PVDF) has emerged as a key polymer for flexible piezoelectric applications due to its intrinsic piezoelectric response, excellent flexibility, and good biocompatibility [6]. The piezoelectricity of PVDF arises mainly from its polar β-phase. Electrospinning, a versatile and effective fabrication method, enables the direct formation of β-phase nanofibers by subjecting polymer jets to strong electric fields and high extensional stresses, thereby suppressing the formation of the non-polar α-phase [7]. Electrospun PVDF-based nanofibers also offer high surface-to-volume ratios and mechanical compliance, which are advantageous for conformal contact with complex body surfaces.

Polyacrylonitrile (PAN), characterized by high tensile strength and thermal stability [8], is often blended with PVDF to improve the mechanical robustness and morphological uniformity of composite fibers. Graphene, owing to its superior electrical conductivity and mechanical strength [9,10], serves as an effective functional nanofiller for enhancing charge transport within the composite structure. In addition, graphene can act as a nucleating agent that promotes β-phase crystallization in PVDF, leading to improved piezoelectric performance [11]. In this work, a lithium phosphate (Li_3_PO_4_) solution was also incorporated to further increase electrical conductivity and support polarization alignment within the polymer matrix.

Recent research has focused on optimizing such polymer-based nanocomposites for advanced sensing applications. Various nanofillers, including carbon nanotubes (CNTs) and other two-dimensional materials, have been explored to maximize piezoelectric output and energy harvesting efficiency [12]. At the same time, considerable effort has been devoted to improving the interfacial compatibility between the polymer matrix and conductive fillers, in order to ensure long-term operational stability and repeatability under dynamic loading conditions [13]. The integration of these enhanced nanocomposites into flexible and skin-conformal substrates has opened new pathways for high-resolution, real-time monitoring of subtle biomechanical signals, such as passive joint movements and arterial pulsations, within the emerging field of wearable and epidermal electronics [1,14].

Despite these advances, several challenges remain for the practical deployment of PVDF-based flexible piezoelectric sensors in passive human motion monitoring. Many reported devices rely on relatively thick films or rigid substrates, which limit conformability and long-term comfort [15]. In other cases, the sensing layers offer good piezoelectric response but suffer from limited electrical conductivity or unstable outputs under low-amplitude mechanical stimuli, such as arterial pulse pressures below 150 mmHg [16,17,18]. Moreover, systematic evaluation of sensor performance using well-defined pressure ranges and custom test platforms is still relatively scarce, making it difficult to compare different material systems and to translate laboratory prototypes into reliable wearable devices [19,20,21].

Previous research has demonstrated that adding graphene to PAN/PVDF-based piezoelectric sensors improves charge transport, electrical conductivity, and interfacial polarisation, which raises output voltage, sensitivity, and signal-to-noise ratio under low-amplitude mechanical stimuli [22,23]. Furthermore, it has been documented that additives based on lithium phosphate encourage dipole alignment and β-phase development in PVDF via ionic interactions, resulting in improved piezoelectric response and signal stability [24]. These results show that lithium phosphate and graphene additions work together to improve electromechanical coupling in PAN/PVDF systems, allowing for more dependable and effective flexible piezoelectric sensors.

In this study, PVDF/PAN/graphene nanocomposite fiber films were fabricated via electrospinning, and their structural, electrical, and piezoelectric properties were systematically characterized. The material design combines PVDF as the piezoelectric matrix, PAN as a mechanical and morphological stabilizer, graphene as a conductive and β-phase-promoting nanofiller, and Li_3_PO_4_ as an additional ionic additive to support charge transport. The sensing performance of the developed films was evaluated through the detection of passive human motion, including finger bending and pulsatile pressure, using custom-designed test platforms capable of applying controlled pressures of 40, 80, and 120 mmHg. The results demonstrate stable millivolt-level outputs with good repeatability and adequate signal-to-noise ratio, highlighting the potential of PVDF/PAN/graphene nanofiber films as flexible piezoelectric sensors for wearable biomechanical monitoring and human–machine interfacing [25].

## 2. Materials and Methods

All chemicals and polymers used in this study were of commercial grade and employed without further purification. Poly(vinylidene fluoride) (PVDF, 99.9%, Sigma-Aldrich) and polyacrylonitrile (PAN, 99.9%, Sigma-Aldrich) served as the primary polymeric components. Dimethylformamide (DMF, 99.9%, Chemsolute) and acetone (99.9%, Sigma-Aldrich) were used as solvents to prepare the spinning solutions. Lithium phosphate (Li_3_PO_4_, 98%, Sigma-Aldrich) was incorporated as an ionic additive to enhance the electrical conductivity of the nanocomposite. Graphene nanoparticles were synthesized from graphite rods by a femtosecond (fs) laser ablation technique and subsequently dispersed in the polymer solutions.

### 2.1. Preparation of PVDF/PAN/Graphene Spinning Solution

The polymer nanocomposite solution was prepared by dissolving 3.0 g of PVDF and 0.2 g of PAN in 20.1 g of DMF under continuous stirring at 70 °C and 400 rpm for 12 h in a covered container to minimize solvent evaporation. After complete dissolution of the polymers was confirmed, 0.02 g of graphene nanoparticles and 0.10 g of Li_3_PO_4_ were added to improve the electrical conductivity of the solution. The mixture was further stirred for 36 h to achieve a homogeneous dispersion of all components, and then allowed to cool to room temperature.

To mitigate undesirable effects such as fiber beading or solution dripping during electrospinning, 1.5 g of acetone was added to the cooled solution. Owing to its higher volatility relative to DMF, acetone facilitates rapid solvent evaporation during fiber formation and supports the development of uniform nanofiber morphology. The acetone-containing solution was stirred for an additional 30 min and subsequently equilibrated at room temperature for 1 h before being loaded into a syringe for electrospinning.

### 2.2. Fabrication of PVDF/PAN/Graphene Nanofiber Films and Sensor Devices

**Figure 1:**
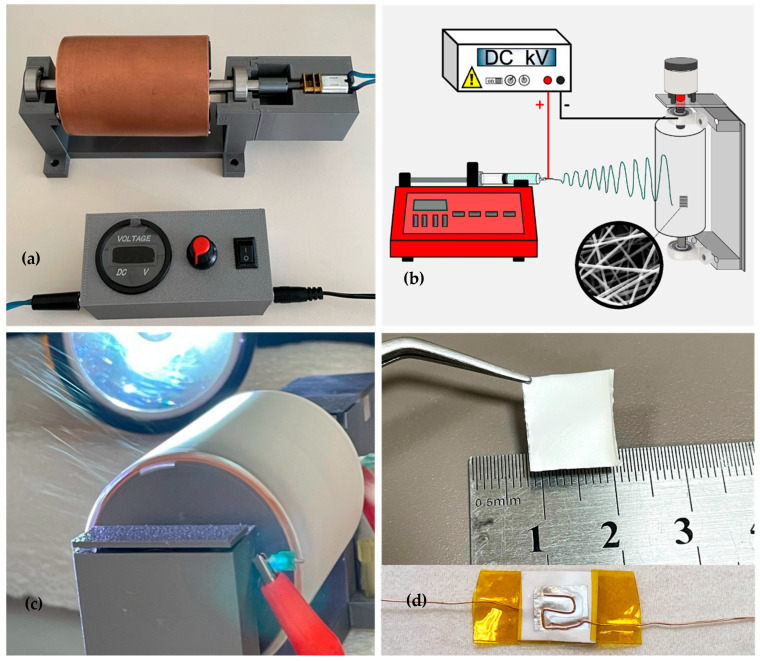
(**a**) Copper cylindrical collector and rotation-speed control unit; (**b**) schematic of the electrospinning setup; (**c**) photograph of the electrospinning process showing nanofiber deposition on the rotating collector; (**d**) fabricated PVDF/PAN/graphene piezoelectric sensor.

Piezoelectric PVDF/PAN/graphene nanofiber films were fabricated using a custom-designed electrospinning system equipped with a cylindrical copper collector, as shown in Figure 1a,b. The setup consisted of a metallic syringe tip (length 1.5 cm) positioned at a controlled distance from the rotating collector. A positive DC high voltage was applied to the needle using a high-voltage power supply, while the collector rotation speed was controlled by an external driver (Figure 1a). A schematic of the overall configuration is presented in Figure 1b, and a photograph of the electrospinning process and nanofiber deposition on the rotating collector is shown in Figure 1c. The final PVDF/PAN/graphene piezoelectric sensor prepared from the nanofiber film is illustrated in Figure 1d.

During electrospinning, nanofibers were deposited onto a cylindrical copper collector with a diameter of 54 mm and a length of 75 mm, rotating at approximately 1500 rpm. The collector was wrapped with 15 µm thick aluminum foil to facilitate fiber collection and subsequent removal of the nanofiber film. Unless otherwise stated, the tip-to-collector distance was set to 18 cm, and a voltage of 18 kV was applied at a position 0.7 cm from the syringe tip. Under these conditions, the target fiber diameter was approximately 200 nm, and the resulting film thickness was maintained between 20 and 25 µm.

To optimize the electrospinning conditions, multiple samples were produced by varying the applied voltage (18, 19, and 20 kV) and the tip-to-collector distance (16, 17, and 18 cm). The sample fabricated at 18 kV with an 18 cm tip-to-collector distance exhibited the most uniform fiber morphology, with minimal bead formation, and was therefore selected for subsequent thermal treatment and sensor fabrication.

Post-fabrication thermal treatment was carried out in a programmable oven to enhance the piezoelectric properties of the nanofiber films. The oven temperature was ramped from room temperature to 130 °C over 1 h, maintained at 130 °C for 3 h, and then allowed to cool back to room temperature within the oven. This annealing process was used to promote the formation of the piezoelectrically active β-phase of PVDF and to stabilize the nanofiber structure.

**Figure 2:**
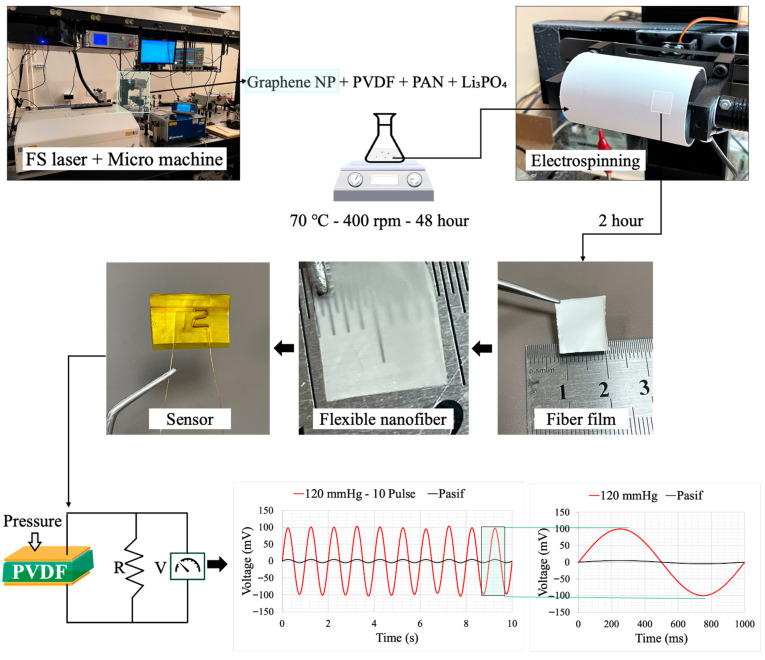
Schematic illustration of the fabrication and testing steps for the PVDF/PAN/graphene piezoelectric sensor: femtosecond (fs) laser system used for graphene nanoparticle synthesis; mixing of graphene NPs with PVDF, PAN, and Li_3_PO_4_ followed by stirring at 70 °C and 400 rpm; electrospinning of the composite solution to obtain flexible nanofiber films; cutting and assembly of the fiber film into a piezoelectric sensor device; and representative output voltage signals under an applied pressure of 120 mmHg for periodic pulse loading and passive deformation.

The overall fabrication and testing workflow for the PVDF/PAN/graphene piezoelectric sensor is summarized in Figure 2. Graphene nanoparticles were first produced by the femtosecond laser system and then mixed with PVDF, PAN, and Li_3_PO_4_ in DMF to form the spinning solution (Figure 2, top row). After stirring at 70 °C and 400 rpm, the solution was electrospun to obtain flexible nanofiber mats. The as-spun mat was peeled from the collector, yielding a free-standing fiber film that could be easily cut and handled, as shown in Figure 2 (middle row).

For sensor assembly, square pieces (10 × 10 mm^2^) were cut from the thermally treated nanofiber films (Figure 2, “Fiber film” and “Sensor”). Aluminum foil was used as the base electrode, onto which a thin layer of silver paste (previously diluted in acetone) was applied to form a conductive interface. Copper wires with a diameter of 0.1 mm were pre-bent into a zigzag pattern and placed on the wet silver layer. They were then fixed in position using Kapton tape to ensure reliable electrical contact. The same procedure was applied to the top surface of the nanofiber film to form a parallel-plate electrode configuration. Although this work used silver paste and aluminium foil for dependable electrical contact and prototyping, these materials may restrict long-term wearability. It has been stated that alternative flexible electrode techniques, like stretchy or textile-integrated electrodes, polymer/graphene composite electrodes, and conductive polymers (like PEDOT: PSS), offer better flexibility, conformability, and durability for wearable applications [26].

Finally, the assembled sensor was subjected to a hot-press treatment at 120 °C. A flat dead weight of approximately 200 g was placed on top of the device for 1 h to ensure good adhesion between layers and stable electrical contact, thereby completing the fabrication of the piezoelectric sensor. The lower part of Figure 2 illustrates the working principle of the device under applied pressure and shows representative output voltage signals under 120 mmHg loading for periodic pulse excitation and passive deformation.

### 2.3. Materials Characterization

The morphology of the electrospun PVDF/PAN/graphene nanofiber films was examined by scanning electron microscopy (SEM, ZEISS EVO LS10, Germany). The crystalline phases and chemical structure of the nanocomposite were analyzed by Fourier transform infrared spectroscopy (FTIR, Bruker Vertex 70, USA). Graphene nanoparticles used in the composite were synthesized with a femtosecond laser system (Quantronix Integra-C, NY, USA).

### 2.4. Electrical Characterization of the Sensor

The electrical response of the PVDF/PAN/graphene sensors was measured using a picoammeter (Keithley 6485) and an digital multimeter (Agilent 34410A). A Python-based graphical interface was written to control the picoammeter and to log nanoampere-level currents during dynamic tests. This interface enabled the adjustment of key settings, including integration time (NPLC), communication speed, and zero-correction, which helped maintain reproducible measurement conditions.

Voltage signals in the millivolt range were recorded in real time with the Agilent 34410A and cross-checked using a digital oscilloscope (LeCroy 9304, NY, USA) equipped with a 10 MΩ, 9.5 pF probe. The excellent agreement between the multimeter and oscilloscope traces confirmed the reliability of the measurement setup.

## 3. Results and Discussion

### 3.1. Morphological Analysis of PVDF/PAN/Graphene Nanofibers

**Figure 3:**
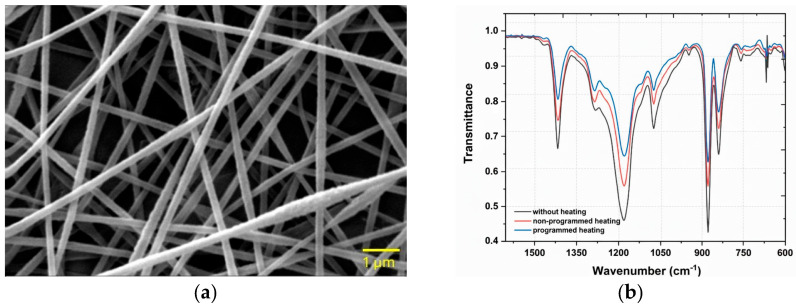
(**a**) SEM image of electrospun PVDF/PAN/graphene nanofibers. (**b**) FTIR spectra of PVDF-based nanofiber films without thermal treatment, with non-programmed heating, and with programmed heating.

The surface morphology and diameter distribution of the PVDF/PAN/graphene nanofiber films were examined by scanning electron microscopy (SEM). The fibers exhibited average diameters in the range of 200–250 nm. As shown in Figure 3a, the nanofiber films produced under the optimized electrospinning conditions exhibit a homogeneous and continuous morphology with no noticeable bead formation. The fiber diameter distribution is narrow and centered around ~200 nm, which provides a high specific surface area beneficial for piezoelectric sensing. Similar diameter ranges and defect-free morphologies have been reported for optimized PVDF-based electrospun nanofibers used in sensing and energy harvesting applications [27,28,29].

The influence of electrospinning parameters, including applied voltage (18, 19, and 20 kV) and tip-to-collector distance (16, 17, and 18 cm), on fiber morphology was also investigated. Higher applied voltages, combined with an appropriate collector distance, reduced the average fiber diameter and yielded smoother, more uniform fibers [30]. This trend is consistent with previous reports on PVDF and other electrospun systems, where increased electric field strength and optimized spinning distance lead to greater jet stretching and finer, bead-free fibers.

### 3.2. FTIR Characterization of PVDF/PAN/Graphene Nanofibers

The crystalline phases of PVDF and the chemical structure of the PVDF/PAN/graphene nanocomposite were analyzed using Fourier transform infrared (FTIR) spectroscopy. The spectra were examined to confirm the presence and relative proportion of the piezoelectrically active β-phase. Characteristic absorption bands at ~840 cm^−1^ and ~1279 cm^−1^ (Figure 3b) are assigned to the β-phase of PVDF, in agreement with previous reports on PVDF-based materials [31,32]. These results indicate that the electrospinning process, together with the inclusion of graphene and Li_3_PO_4_, effectively promotes the transformation from the non-polar α-phase to the electroactive β-phase.

Graphene acts as a nucleating agent for β-phase crystallization by providing high-surface-area interfaces that interact with the PVDF chains, leading to enhanced dipole alignment and electroactive phase content without requiring strong mechanical stretching [33]. PAN mainly contributes to the structural integrity and mechanical properties of the composite, improving the flexibility of the nanofibers.

The effects of thermal treatment on phase formation were also investigated. FTIR analysis revealed that the programmed heating and cooling cycles significantly increased the β-phase content, as indicated by the intensified band at ~840 cm^−1^ and the attenuation of α-phase bands, such as those near 764 and 875 cm^−1^, in agreement with previous reports on annealed PVDF-based films and nanofibers [34,35]. A shoulder or band in the 870–880 cm^−1^ region is associated with CH_2_ rocking and C–C skeletal vibrations and is often linked to less ordered or more amorphous segments of the PVDF-based matrix, which contribute to the overall flexibility of the fibers.

### 3.3. Custom Bending Test Device

**Figure 4:**
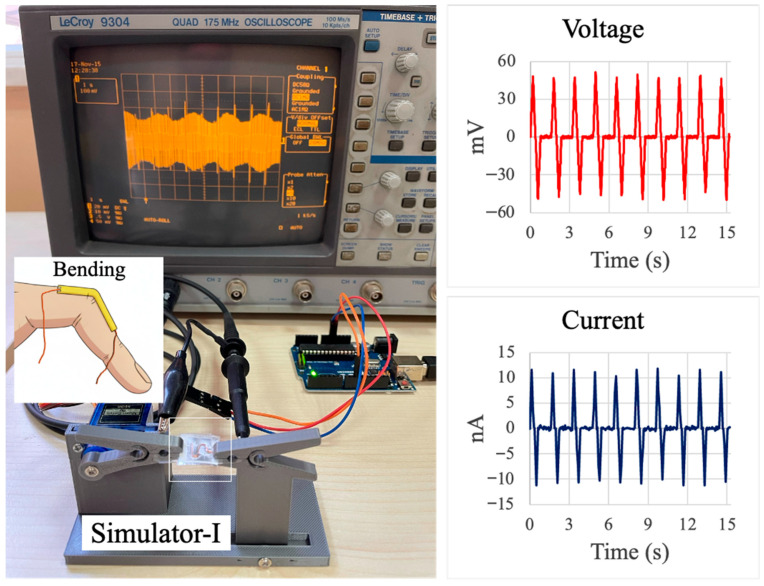
Bending–releasing test system for the PVDF/PAN/graphene piezoelectric sensor. (**Left**): photograph of the custom Simulator-I platform with the sensor clamped between two movable arms and connected to a LeCroy 9304 oscilloscope; the inset illustrates the intended placement of the sensor along a finger joint. (**Right**): representative output voltage (**top**) and current (**bottom**) waveforms recorded over 15 s during periodic bending–unbending cycles, showing stable, repeatable peaks associated with each deformation event.

The bending–releasing test platform, shown in Figure 4, was used to quantify the electromechanical response of the PVDF/PAN/graphene sensor under controlled, repeatable deformation. The sensor was mounted between the two arms of the Simulator-I rig, which are driven by a servo motor so that one end of the sensor is fixed while the other is periodically displaced. This motion reproduces the curvature that would occur when the device is attached along a finger joint, as illustrated by the schematic inset in Figure 4. The bending frequency and amplitude were set to mimic slow, passive finger movements typically encountered in daily activities. The photograph in Figure 4 shows the complete measurement setup, with the oscilloscope displaying the real-time voltage waveform generated by the sensor’s cyclic bending.

The right-hand panels of Figure 4 present representative voltage and current responses over a 15 s interval. The voltage trace exhibits a series of sharp, alternating positive and negative peaks corresponding to the bending and releasing phases of each cycle, with peak magnitudes on the order of several tens of millivolts. The current trace shows a similar periodic pattern with amplitudes in the nanoampere range. The regularity of the peaks and the stable baseline indicate good repeatability of the sensor response under cyclic loading, confirming that the PVDF/PAN/graphene nanofiber film can reliably convert low-amplitude mechanical bending into measurable electrical signals suitable for passive motion monitoring.

**Figure 5:**
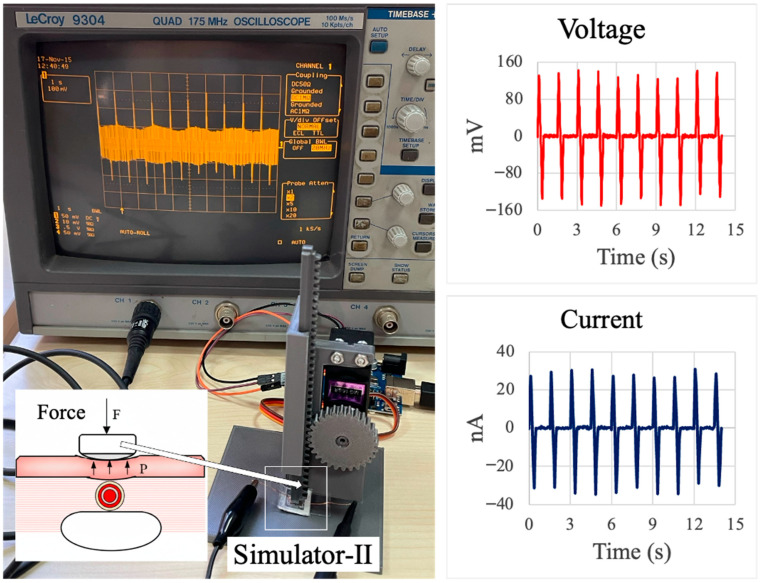
Force test system (Simulator-II) for evaluating the PVDF/PAN/graphene piezoelectric sensor under pulse-like loading. (**Left**): photograph of the setup with the sensor mounted at the base of a rack-and-pinion mechanism driven by a servo motor and connected to a LeCroy 9304 oscilloscope; the inset illustrates the application of a normal force on the sensor placed above a superficial artery. (**Right**): representative output voltage (**top**) and current (**bottom**) waveforms recorded over 15 s during periodic force loading, showing stable, repeatable peaks suitable for simulating arterial pulse pressure.

To assess the sensor response to localized, pulse-like loading, a second test apparatus (Simulator-II) was constructed, as shown in Figure 5. In this setup, the sensor is mounted vertically on a rigid support and periodically pressed by a plunger driven by a servo motor through a rack-and-pinion mechanism. The schematic inset in Figure 5 illustrates the intended configuration for biomedical use, where the device is placed on the skin above a superficial artery and subjected to small, periodic, normal forces corresponding to pulsatile blood pressure.

The servo motor and linear motion of the plunger enable well-defined loading–unloading cycles with adjustable amplitude and frequency, allowing controlled simulation of pulse pressures in a laboratory environment. The oscilloscope photograph in Figure 5 shows the real-time voltage waveform generated by the sensor during repeated normal loading. The right-hand plots display representative voltage and current responses over a 15-s period. The voltage signal exhibits sharp, alternating peaks with magnitudes approaching ±150 mV, while the corresponding current waveform reaches amplitudes of several tens of nanoamperes. The regularity and stability of these peaks demonstrate that the PVDF/PAN/graphene sensor can reliably detect low-amplitude, periodic normal forces that mimic arterial pulse pressure, without the need for human subjects during calibration and optimization.

The output level is consistent with other PVDF-based pressure sensors operating in a similar force range. Chamankar et al. reported a flexible PVDF film pressure sensor that produced an open-circuit voltage of 184 mV under an applied force of 2.125 N [36]. Zhang et al. summarized PVDF/ZnO and PVDF/G-ZnO nanofiber devices with peak voltages between 1.7 and 4.4 V, but these values were obtained under higher compressive forces of around 5 N [37]. Chen et al. further demonstrated PVDF/GO pressure sensors delivering up to 4.93 V at an impact force of 120 N [38]. Compared with these systems, the PVDF/PAN/graphene sensor in this work is tested under much lower, pulse-like pressures designed to emulate arterial loading rather than impact or tapping forces. As a result, the absolute voltage is lower, but the signal is still easily measurable with standard high-impedance electronics, and no ceramic fillers or complex multilayer structures are required. This places the present device among simple, flexible PVDF-based sensors that are suitable for passive monitoring of subtle physiological forces.

**Figure 6:**
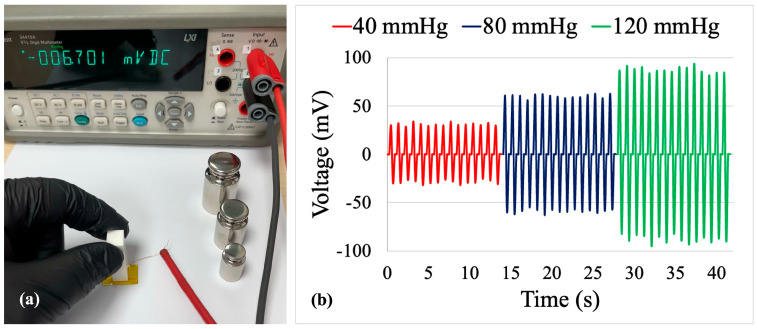
(**a**) Experimental setup for DC voltage characterization of the PVDF/PAN/graphene sensor using an Agilent 34410A multimeter with 1 µV resolution and a calibrated dead-weight set. (**b**) Voltage responses of the sensor under applied pressures of 40, 80, and 120 mmHg, showing the increase in peak amplitude with increasing pressure.

The sensor active area is precisely defined as 10 × 10 mm^2^ (0.01 m^2^). Dead weights corresponding to pressures of 40, 80, and 120 mmHg were approximately 54 g, 109 g, and 163 g, respectively, yielding applied forces of 0.533 N, 1.067 N, and 1.600 N. The pressure–voltage relationship exhibits a linear fit with R^2^ ≈ 0.98, confirming consistent stress transfer across the loading range. The signal-to-noise ratio (SNR) was quantified from oscilloscope traces (Figure 6b) as SNR (dB) = 20 × log_10_(V_peak_/V_noise_). Baseline noise measured approximately 1.5–2.0 mV RMS, yielding SNR values of 24 dB (40 mmHg), 30 dB (80 mmHg), and 34 dB (120 mmHg), which are suitable for wearable biomedical applications. Film thickness variation (20–25 μm, ±12.5%) introduces an estimated pressure calibration uncertainty of ±5–10%.

### 3.4. Pressure–Voltage Measurement

For quantitative calibration, the sensor electrodes were connected to an Agilent 34410A multimeter operated in DC voltage mode with a resolution of 1 µV (Figure 6a). Three distinct pressure levels were applied to the active area of the device using a calibrated dead-weight set. The corresponding pressures were calculated according to the DKD-R 3-3 guideline for force-measuring devices, and the local gravitational acceleration was obtained from the g-Extractor tool provided by Physikalisch-Technische Bundesanstalt (PTB).

Figure 6b shows representative time-domain voltage signals recorded at 40, 80, and 120 mmHg, which span the typical physiological range of arterial blood pressure. In each case, the sensor generates periodic peaks whose amplitude increases with applied pressure. The average peak voltages were 25–30 mV at 40 mmHg, 53–60 mV at 80 mmHg, and 80–90 mV at 120 mmHg. The nearly proportional increase in peak amplitude with pressure indicates an approximately linear response, corresponding to an effective sensitivity of about 0.7–0.8 mV mmHg^−1^ in this range. The waveforms remain stable over repeated cycles, with only minor fluctuation in peak height, confirming good reproducibility and an adequate signal-to-noise ratio for direct readout with standard high-impedance electronics.

### 3.5. Performance Evaluation of the Piezoelectric Sensor

The developed PVDF/PAN/graphene piezoelectric sensor exhibited rapid and stable electrical responses to different types of mechanical stimuli (Table 1). Under bending–releasing excitation and pulse-like normal loading (Figure 4 and Figure 5), the device generated clear, alternating voltage peaks with amplitudes of several tens to more than one hundred millivolts and corresponding currents in the nanoampere range, exhibiting good cycle-to-cycle repeatability. In the calibrated dead-weight tests (Figure 6), the average peak voltages increased from 25–30 mV at 40 mmHg to 53–60 mV at 80 mmHg and 80–90 mV at 120 mmHg, confirming a pressure-dependent response in the physiological blood-pressure range. The voltage–time traces accurately follow the frequency and rhythm of the applied stress–relaxation cycles, and the signal-to-noise ratio is sufficient for direct acquisition with standard high-impedance electronics, indicating that the fabricated sensors are suitable for wearable biomedical monitoring of low-amplitude mechanical signals.

To contextualize the performance of the present PVDF/PAN/graphene/Li_3_PO_4_ sensor within the broader research landscape, a comprehensive comparison with recently reported PVDF-based piezoelectric sensors is presented in Table 1. The present sensor is uniquely specialized for low-pressure physiological monitoring (40–120 mmHg), a clinically relevant pressure range for arterial pulse and respiration detection. While many PVDF-based sensors achieve higher absolute voltage outputs through the use of ceramic fillers (BaTiO_3_, PZT) or heterostructured composites (ZnO nanorods with graphene), they typically operate within much higher pressure or force ranges (0.5–120 N, corresponding to 5–1200 kPa), far exceeding physiological blood pressures.

The comparative analysis reveals that the present work addresses a significant gap: while many sensors achieve high voltages in high-pressure regimes, few are systematically optimized for the subtle, physiologically relevant pressure range of arterial pulse monitoring (40–120 mmHg). By combining fine nanofiber morphology, physiologically matched sensitivity, a simple material composition (graphene without ceramic fillers), and scalable electrospinning fabrication, this work provides a practical and cost-effective platform for wearable biomedical monitoring of arterial pulse and respiratory signals.

The output level demonstrated here (80–90 mV at 120 mmHg) is suitable for physiological measurements and can be easily measured with standard high-impedance electronics, without the need for ceramic fillers or complex multilayer structures. This positions the device as a practical and flexible sensor suitable for passive monitoring of subtle physiological forces, representing a meaningful advancement toward deployable, wearable biomedical devices for real-world clinical applications.

## 4. Conclusions

In this work, flexible piezoelectric sensors based on electrospun PVDF/PAN/graphene nanofiber films were designed, fabricated, and systematically evaluated for passive monitoring of human motion and pulse-like pressure. A PVDF/PAN/graphene/Li_3_PO_4_ spinning solution was optimized to produce uniform, bead-free nanofibers with diameters of 200–250 nm and film thicknesses of 20–25 µm. SEM imaging confirmed the homogeneous nanofiber morphology, while FTIR analysis showed the presence of a high fraction of the electroactive β-phase of PVDF, further enhanced by programmed thermal treatment.

The nanofiber films were integrated into compact parallel-plate sensor structures and characterized using custom-built bending and force simulators. Under cyclic bending and pulse-like normal loading, the sensors generated stable, repeatable voltage peaks of several tens to more than one hundred millivolts, with corresponding nanoampere-level currents. Calibration with a dead-weight setup in the range 40–120 mmHg yielded average peak voltages of 25–30 mV, 53–60 mV, and 80–90 mV, corresponding to an effective sensitivity of approximately 0.7–0.8 mV mmHg^−1^. The signals faithfully followed the frequency and rhythm of the applied mechanical stimuli and remained stable over repeated cycles, demonstrating good reproducibility and an adequate signal-to-noise ratio for direct readout.

Overall, the results demonstrate that electrospun PVDF/PAN/graphene nanofiber films, when combined with simple post-processing and device assembly steps, provide an effective platform for flexible piezoelectric sensors capable of detecting low-amplitude mechanical events within the physiological pressure range. The proposed approach offers a straightforward route towards lightweight, conformal devices for wearable biomechanical monitoring and pulse sensing, and can be extended to other nanofiber architectures and transduction schemes in future work.

## Figures and Tables

**Table 1:** Comparative Performance of Recent PVDF-Based Piezoelectric Sensors. This table positions the PVDF/PAN/graphene/Li_3_PO_4_ sensor developed in this work within the broader landscape of recent PVDF-based wearable piezoelectric devices. The present work is uniquely optimized for low-pressure physiological monitoring (40–120 mmHg). Alternative approaches employ ceramic fillers (BaTiO_3_, PZT), heterostructured composites (ZnO nanorods), or advanced morphology engineering (porous nanofibers) to achieve performance across different pressure/force ranges and application domains. Key distinguishing features of the present work include the finest nanofiber diameter (200–250 nm), the thinnest film (20–25 μm), the simplest material composition without ceramic fillers, and scalable electrospinning with femtosecond laser-synthesized graphene nanoparticles.

Material Composition	Fabrication Method	Year	Fiber Diameter (nm)	Film Thickness (μm)	Peak Voltage	Sensitivity	Operating Range	Target Application	Reference
PVDF/PAN/Graphene/Li_3_PO_4_	Electrospinning + fs-laser graphene	2025	200–250	20–25	80–90 mV @ 120 mmHg	0.7–0.8 mV/mmHg	40–120 mmHg (physiological)	Pulse monitoring, wearable biomedical	This work
PVDF-TrFE + 3 wt% BaTiO_3_	Electrospinning	2025	200–300	43	9800 mV @ 16 kPa	370 mV/kPa (6.4–16 kPa)	6.4–16 kPa	Arterial pulse, wireless wearable	[16]
Graphene-doped PVDF (1 wt%)	Electrospinning	2020	400–600	30–50	2000–3000 mV	0.006 V/° (angular)	Bending 120–60°, low pressure	Gesture recognition, motion tracking	[22]
Porous Graphene/PVDF (0.1 wt%)	Electrospinning + spinodal decomposition	2021	500–800	40–60	1500–2000 mV	Not specified	Low pressure, motion sensing	Biocompatible wearable	[30]
PVDF/ZnO nanorods-Graphene	Electrospinning	2023	300–500	35	1700–4400 mV @ 5 N	0.34–0.88 V/N	Medium–high pressure (up to 5 N)	Motion sensing, nanofiber devices	[37]
PVDF/Graphene Oxide (GO)	Electrospinning	2022	250–450	30–40	4930 mV @ 120 N	0.041 V/N	High-force impact (120 N)	Joint monitoring, impact sensing	[38]
PVDF + PZT nanoparticles	Solution mixing/casting	2020	N/A	N/A	184 mV @ 2.125 N	86.5 mV/N	Impact/pressure (0–2.125 N)	Energy harvesting, pressure sensor	[39]

## Data Availability

The datasets generated and/or analysed during the current study are included in this published article. Further data are available from the corresponding author upon reasonable request.
